# 1703. Pseudo-Outbreak of Methicillin-Resistant *Staphylococcus aureus* in a Neonatal Intensive Care Unit: Implications for Surveillance

**DOI:** 10.1093/ofid/ofad500.1536

**Published:** 2023-11-27

**Authors:** Ashley Dauphin, Patrick S Gordon, Gina L Story, Munish Gupta, Jordan Schultz, Matthew Doucette, Melissa Cumming, Lorinda Longhi, James Kirby, Preeti Mehrotra

**Affiliations:** Beth Israel Deaconess Medical Center, Boston, Massachusetts; Beth Israel Deaconess Medical Center, Boston, Massachusetts; BIDMC, Melrose, Massachusetts; Beth Israel Deaconess Medical Center, Boston, Massachusetts; Massachusetts Department of Public Health, Jamaica Plain, Massachusetts; Massachusetts Department of Public Health, Jamaica Plain, Massachusetts; Massachusetts Department of Public Health, Jamaica Plain, Massachusetts; Beth Israel Deaconess Medical Center, Boston, Massachusetts; Beth Israel Deaconess Medical Center, Boston, Massachusetts; Beth Israel Deaconess Medical Center/Harvard Medical School, Boston, Massachusetts

## Abstract

**Background:**

We conduct weekly surveillance screening for methicillin-resistant *Staphylococcus aureus* (MRSA) in our neonatal intensive care unit (NICU). In September 2021, we noted an increase in positive MRSA screening results and identified 2 patients with MRSA wound infection. We conducted an investigation to explore the possibility of in-unit transmission.

**Methods:**

We launched a multi-pronged investigation that included additional patient screening. Surveillance isolates were assessed for the presence of MRSA using chromogenic agar. Additional antimicrobial sensitivity testing was performed on an automated bacterial identification system if chromogenic agar results were inconclusive, fig 1 & 2. We also conducted audits of staff hand hygiene, enhanced cleaning and disinfection of shared patient-care equipment, implemented standardized chlorhexidine gluconate (CHG) scrub use for NICU staff upon unit entry, and added new CHG bathing for certain patients.

Positive control chromogenic agar plate

**Figure:**
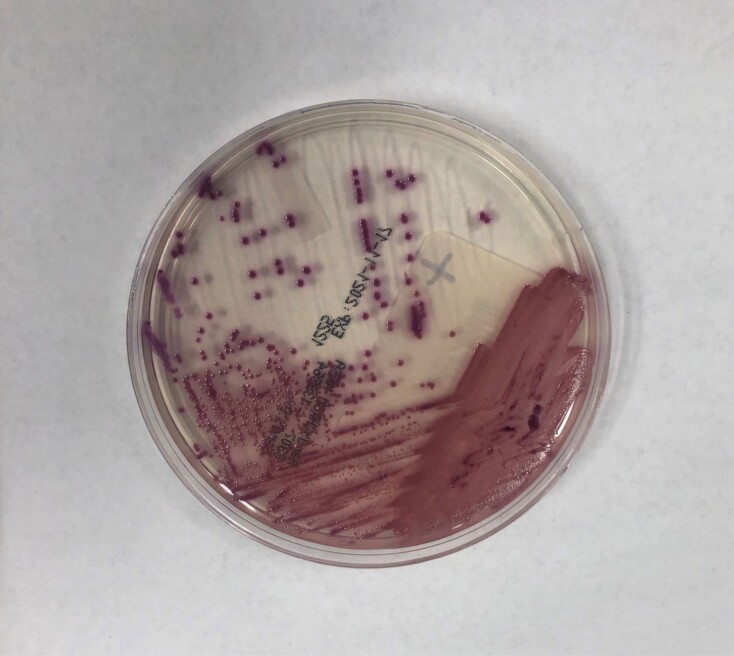

Ambiguous chromogenic agar results

**Figure:**
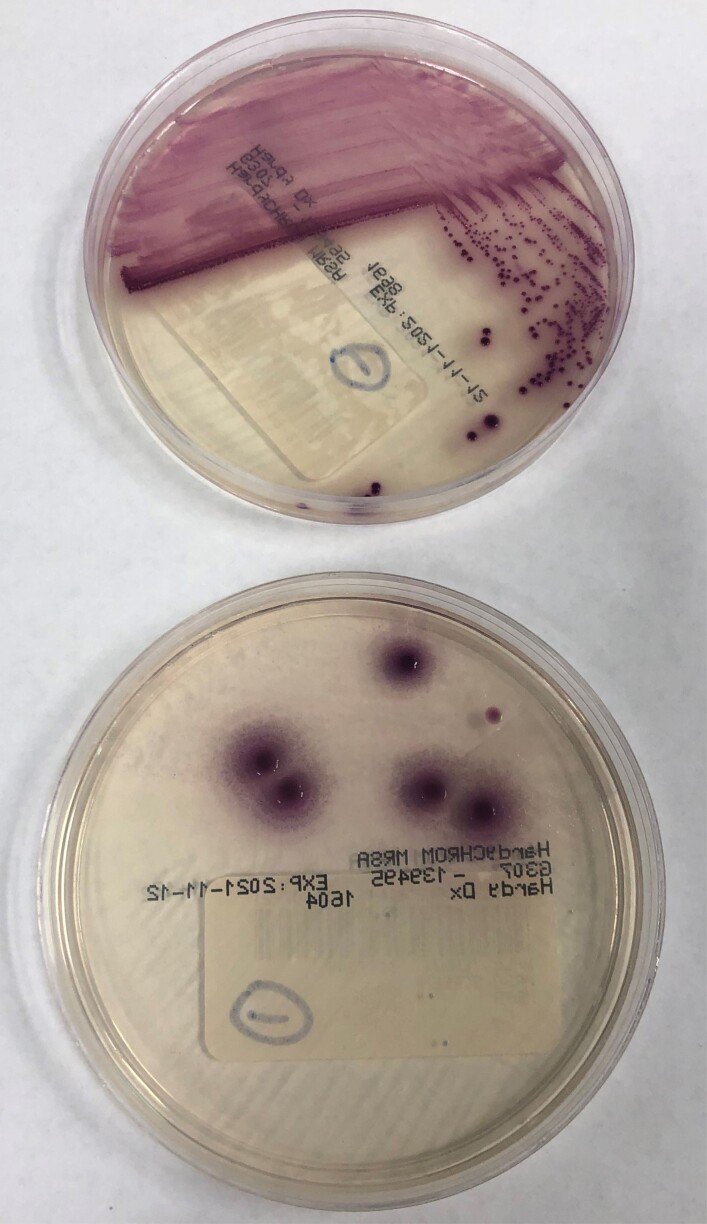

**Results:**

Over the course of a 6-week long investigation, 18 neonates were found to be MRSA-positive on surveillance swabs using chromogenic agar. In the first 2 weeks of the investigation, PFGE showed that 9 MRSA isolates were genetically distinct. Over the next weeks, additional MRSA-positive surveillance swabs were collected and ultimately 15 isolates underwent whole genome sequencing at the Mass. State Public Health Laboratory. Only 2 pairs of isolates appeared related. Antimicrobial resistance genes were identified and revealed only 6 of the 15 isolates contained the *mecA* gene consistent with MRSA. As a result, all 18 positive surveillance swabs obtained during the investigation underwent full identification and sensitivity testing confirming only 6 samples contained MRSA and 12 contained methicillin-sensitive *Staphylococcus aureus* (MSSA). Testing discordance with breakthrough MSSA growth was reported to the chromogenic agar manufacturer.

**Conclusion:**

Our investigation identified that an increase in MRSA-positive surveillance swabs was a pseudo-outbreak due to false-positive MSSA growth on MRSA selective media. At the conclusion of this investigation, the need for ongoing surveillance swabs was re-evaluated and ultimately discontinued. Clinical NICU specimens are now monitored in real-time by Infection Control.

**Disclosures:**

**James Kirby, MD, D(ABMM)**, Abbott Molecular: Educational Grant

